# New Technological Approaches in Occupational Therapy for Pediatric Cerebral Palsy: A Systematic Review

**DOI:** 10.3390/healthcare13050459

**Published:** 2025-02-20

**Authors:** Lara Martínez-Rodríguez, Cristina García-Bravo, Sara García-Bravo, María Salcedo-Pérez-Juana, Jorge Pérez-Corrales

**Affiliations:** 1 Department of Physical Therapy, Occupational Therapy, Physical Medicine and Rehabilitation, Universidad Rey Juan Carlos, 28922 Alcorcón, Spain; l.martinezro.2023@alumnos.urjc.es; 2 Department of Physical Therapy, Occupational Therapy, Physical Medicine and Rehabilitation, Research Group of Humanities and Qualitative Research in Health Science (Hum&QRinHS), Universidad Rey Juan Carlos, 28922 Alcorcón, Spain; maria.perezjuana@urjc.es (M.S.-P.-J.); jorge.perez@urjc.es (J.P.-C.); 3 Physiocare Madrid, Physiotherapy Clinic, 28026 Madrid, Spain; sara.garcia.bravo@urjc.es

**Keywords:** cerebral palsy, occupational therapy, technology, rehabilitation

## Abstract

Background/Objectives: Pediatric cerebral palsy (CP) is a neurological disorder that affects motor skills, posture, and muscle coordination, impacting children’s daily functioning and quality of life. Treatment approaches in occupational therapy aim to enhance motor function and functional independence through a variety of rehabilitative techniques. Recently, new technologies—such as virtual reality, robotics, and assistive devices—have emerged as promising tools in occupational therapy to complement traditional interventions and potentially enhance motor and sensory outcomes in children with CP. This systematic review aims to analyze the effectiveness of these innovative technological interventions in the rehabilitation of children with CP. Methods: A systematic review was conducted and different scales were used to assess the risk of bias and methodological quality. The degree of evidence and level of recommendation were established according to the Grading of Recommendations Assessment, Development, and Evaluation (GRADE). The review adhered to PRISMA guidelines, with a comprehensive literature search performed across multiple databases, including Cochrane, Web of Science, and PubMed. This systematic review has been registered in the PROSPERO database with the registration number CRD42025630162. Results: The reviewed studies indicate that technologies such as functional electrical stimulation, robotic assistance, and adaptive devices have shown improvements in mobility, motor control, posture, strength, and autonomy in children with CP. Similarly, virtual environments support the correction of facial dysfunctions and enhance social interaction through video games and social networks. While these tools hold significant potential for rehabilitation, further adjustments are needed to achieve more consistent results. Conclusions: Emerging technologies appear to be effective tools in occupational therapy for pediatric CP, with positive impacts on motor skills and functional capabilities. Nevertheless, further studies with larger sample sizes and rigorous methodological designs are necessary to confirm these findings and establish more robust evidence on their long-term efficacy.

## 1. Introduction

Currently, cerebral palsy (CP) is one of the most prevalent conditions among children and remains the leading cause of childhood disability. It encompasses a group of chronic disorders affecting movement and posture, resulting in activity limitations. These disorders arise from non-progressive abnormalities, disruptions, or injuries to the developing brain, primarily occurring during the early stages of brain development [1].

The prevalence of CP has remained stable over the past decade, affecting approximately 2.1 children per 1000 live births, with similar rates reported in Europe, the United States, Australia, and Asia. However, a notably higher incidence is observed among preterm infants born before 28 weeks of gestation (111.8 per 1000 live births) and in those with a birth weight below 1500 g (59.2 per 1000 live births) [1,2].

CP can be classified into various subtypes, with spastic CP being the most prevalent. This subtype is characterized by increased muscle tone in the extremities, leading to spasticity and scissoring of the lower limbs. Within spastic CP, specific forms include quadriplegia, diplegia, hemiplegia, paraplegia, monoplegia, and triplegia. The second most common subtype is extrapyramidal CP, also known as athetoid CP, which typically presents with hypotonia during early infancy, progressing to choreoathetoid movements and dystonia in older children. Ataxic CP is marked by hypotonia and difficulties with coordination, while mixed CP involves a combination of features from different clinical subtypes [3,4,5].

The etiology of CP is multifactorial, with triggers often difficult to pinpoint. These causes are commonly categorized as genetic, prenatal, perinatal, or postnatal. Prenatal factors include brain dysgenesis, cerebral malformations, intrauterine hemorrhage, and congenital infections such as cytomegalovirus, rubella, and toxoplasmosis. Perinatal causes are primarily related to perinatal asphyxia and birth trauma. Postnatal factors include intraventricular or subependymal hemorrhages, meningitis/encephalitis, non-accidental head trauma, symptomatic hypoglycemia, hydrocephalus, and hyperbilirubinemia [3,4].

CP significantly impacts motor abilities, particularly muscle tone and voluntary movement control. These factors are critical for the effective use of extremities, as they must respond to and follow instructions from the central nervous system. When these abilities fail to meet movement demands, functionality in the affected body parts is compromised. Additionally, postural issues further influence functionality, as limited posture can hinder the operation of other body parts due to their interdependence [6,7,8,9]. Beyond motor impairments, individuals with CP often experience varied functional limitations, including difficulties with mobility, communication, swallowing, feeding, and cognitive development. These challenges, related to the severity of symptoms, can substantially hinder everyday functioning and interaction with the environment [9]. Children with CP often face challenges in postural control that affect their motor activity and interaction with their environment. To maintain stability, they adopt compensatory strategies, which may lead to imbalances and deformities. Balance development is closely related to motor, sensory, social, and emotional skills. Achieving balance reactions requires proper head control and adequate muscle tone, but in CP, these processes may be delayed or unattained, hindering key motor milestones such as walking. The visual, vestibular, and somatosensory systems, which are essential for maintaining balance, may also be affected by delays in psychomotor development [9,10,11].

Sensory challenges in CP, such as difficulties in tactile discrimination and bimanual coordination, further compromise functional abilities. Proprioception, the ability to perceive body position, plays a critical role in sensory–motor integration. Impaired proprioception can disrupt the execution of tasks and postural control. For instance, a lack of body position awareness can impede precise movements and proper postural adjustments [12].

Cognitive difficulties associated with CP often affect attention, memory, comprehension, perception, and executive functions, as well as agnosias and praxis. These challenges can negatively impact communication and linguistic abilities, leading to difficulties in social interactions [13].

Given the wide range of areas affected by CP, a multidisciplinary team is essential in the rehabilitation process. This team should include specialists such as rehabilitation physicians, physiotherapists, occupational therapists, speech therapists, neuropsychologists, social workers, and others [9,13]. Occupational therapy (OT) is a key discipline in CP rehabilitation. According to the World Federation of Occupational Therapists (WFOTs), OT is a health profession centered on the patient, with the primary goal of promoting health and well-being through engagement in meaningful occupations. Occupational therapists work with individuals and communities to enhance their ability to participate in daily activities they want, need, or are expected to perform. This may involve adapting occupations or the environment to facilitate active participation [14].

The role of occupational therapy in supporting the functional engagement of children with CP is vital, as it focuses on enabling individuals to maintain their roles and develop skills to achieve the highest level of participation and self-determination within their environments. Integrating new technologies into this discipline could provide a more holistic approach to intervention. Information and communication technologies (ICTs), new technologies, and digital technologies have been developed to support, monitor, and enhance well-being, as well as to assist professionals in this complex task [15,16].

ICT encompasses a range of tools and techniques used to process and transmit information via computing, the internet, and telecommunications. These technologies facilitate learning and skill development by enabling the exchange of information in innovative ways. Today, ICT is utilized to promote health and prevent diseases, strengthening care processes in diagnosis, treatment, and prognosis through updated and innovative programs and services [16,17,18].

Consequently, conventional therapies aimed at improving the quality of life for individuals with CP have begun integrating new and digital technologies or ICT as an additional tool to their traditional approaches [18,19,20]. Several disciplines are increasingly incorporating new and digital technologies as complementary tools in rehabilitation treatments, yielding positive outcomes [16,17,18,19,20,21,22]. Currently, studies investigating the use of new technologies as adjuncts to CP rehabilitation span various fields [20,21]. A significant portion of this research focuses on evaluating the efficacy of video game-based therapy in rehabilitation [17,20,21,22]. However, there is a notable scarcity of studies specifically dedicated to exploring the benefits of integrating new technologies or ICTs into occupational therapy interventions for CP.

The purpose of this study is to conduct a systematic review of the available data on the application of new and digital technologies or ICT in occupational therapy for CP treatment. The aim is to evaluate how these technologies impact patients and determine whether they contribute to improving their autonomy.

## 2. Materials and Methods

### 2.1. Search Methodology

We conducted a systematic review of studies examining the use of new technologies in occupational therapy to enhance autonomy in children with CP. To guide the research process, a focused question (RQ1) was formulated using the PICO framework [23]: “Which emerging technologies, including digital and other new technologies, are currently utilized in occupational therapy for children with CP?” Additionally, a second question (RQ2) was posed: “Does occupational therapy incorporating new technologies improve autonomy in children with CP?” Based on these questions, relevant search terms were defined and verified for inclusion in the Medical Subject Headings (MeSHs) database. Primary and entry terms were selected for database searches, and Boolean operators such as “AND” and “OR” were used to refine the search strategy.

The keywords employed included the following: “Pediatric Cerebral Palsy”, “Cerebral Palsy”, “Child”, “Infant”, “Adolescent”, “Pediatrics”, “Occupational Therapy”, “Activities of Daily Living”, “Independence”, “Functional Independence”, “New Technologies”, “Assistive Technology”, “Emerging Technologies”, “Technology”, “Rehabilitation Technology”, “Information and communication technologies”, “Virtual Reality”, and “Robotics”.

**Figure 1:**
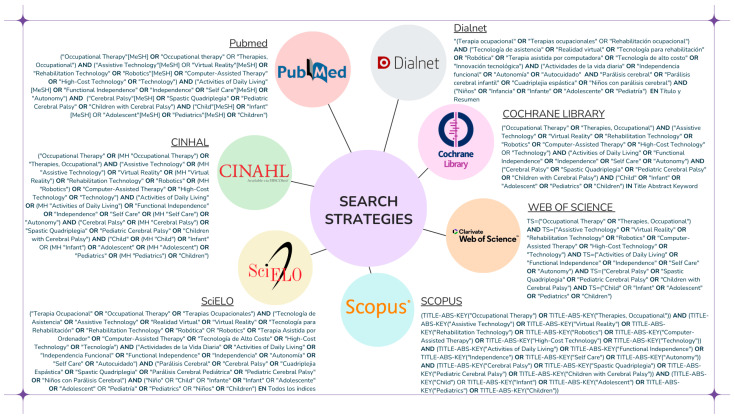
Search strategies.

The initial article search was conducted in the following databases: PubMed, Cochrane Database of Systematic Reviews, CINAHL, Scopus, Web of Science, SciELO, and Dialnet. The search was performed between April 2024 and February 2025, with strategies tailored to each database (Figure 1).

The selection of keywords for this study was carefully structured to align with a taxonomic and functional view of assistive technology, as outlined in the ISO 9999 Assistive Products—Classification and Terminology Standard [18]. This standard classifies assistive products based on the activities and participation they support, rather than solely on their technological attributes. Consequently, our keyword selection reflects not only emerging technological advancements but also their role in promoting independence and participation in pediatric rehabilitation, specifically for children with CP.

First, terms such as “Activities of Daily Living”, “Independence”, and “Functional Independence” were chosen to align with the ISO 9999:2022 framework, as they capture the fundamental purpose of assistive technologies—enhancing functional autonomy and improving quality of life [18]. These terms ensure that the classification of assistive technology is understood beyond a purely technological perspective and is instead considered in the context of its practical application in rehabilitation and occupational therapy.

Second, given the focus of our study on new and emerging assistive technologies, we included terms such as “New Technologies”, “Emerging Technologies”, “Technology”, “Rehabilitation Technology”, “Information and Communication Technologies”, “Virtual Reality”, and “Robotics”. These keywords were selected based on their increasing presence in assistive technology research and their relevance in pediatric rehabilitation. While ISO 9999:2022 [18] does not explicitly differentiate “new” assistive technologies, our selection reflects terminology widely used in the contemporary literature [24,25] to describe technological innovations in the field.

Third, to ensure specificity in addressing pediatric CP, we incorporated population-specific terms such as “Pediatric Cerebral Palsy”, “Cerebral Palsy”, “Child”, “Infant”, “Adolescent”, and “Pediatrics”. These keywords allow for a targeted approach to understanding the role of assistive technology in this population.

Finally, the inclusion of “Occupational Therapy” reflects the essential role of this field in the assessment, prescription, and implementation of assistive technologies. Occupational therapists play a key role in supporting activities of daily living (ADLs) and fostering functional independence in children with CP, making this keyword integral to our study’s scope.

In summary, our keyword selection was guided by a balance between technological innovation, user functionality (ISO 9999:2022 alignment), and clinical relevance in pediatric rehabilitation. By mapping our findings to ISO 9999:2022 and referencing the previous literature, we provide a structured and comprehensive framework for understanding how assistive technology contributes to functional independence in children with CP.

This systematic review included scientific articles published between 1 January 2010, and 31 December 2024. The language of publication was restricted to Spanish or English. This approach ensured the collection of relevant and up-to-date information on the topic of interest, providing a robust foundation for the advancement of clinical research.

### 2.2. Inclusion Criteria

The following inclusion criteria were established to determine eligibility for participation in the studies: the search was limited to randomized and non-randomized clinical trials that examined the impact of various interventions on the pediatric population with CP at any stage of the condition; articles published between 1 January 2010, and 31 December 2024; and publications in English or Spanish. Only articles published in scientific journals were considered valid, provided they addressed the topic of robotic therapy or new technologies in the treatment of CP.

The exclusion criteria were also defined to ensure the inclusion of only pertinent results. Systematic reviews, letters to the editor, duplicate publications, and opinion articles lacking scientific rigor were excluded. Additionally, experimental studies involving animals were omitted, as they do not directly reflect the clinical context of CP.

These inclusion and exclusion criteria ensure that the selected studies are relevant, reliable, and contribute meaningfully to advancing knowledge and treatment of CP in the pediatric population.

### 2.3. Article Selection and Evaluation of Methodological Quality

The search for information was conducted in the databases mentioned earlier, using the predefined strategy tailored to each specific database. Duplicate articles and records marked as ineligible by automation tools were subsequently removed, with Mendeley utilized for this purpose. The titles and abstracts of the remaining studies were reviewed, excluding those unrelated to the topic or with unsuitable designs. Information was collected in a standardized manner, adhering to the Consolidated Standards of Reporting Trials (CONSORTs) Statement [26]. Finally, the full texts of the remaining publications were analyzed, and those not meeting the selection criteria were excluded. Two authors independently reviewed the full texts of the selected articles and organized them according to their relevance. The following information was extracted from each publication: study objectives, variables studied, study design and description, randomization and masking, inclusion and exclusion criteria, intervention description, materials used, and results. Any discrepancies during data extraction were resolved by a third investigator.

To assess the risk of bias and methodological quality of the included studies, validated tools tailored to each study design were applied. For randomized controlled trials (RCTs), the PEDro scale was used, evaluating key aspects such as randomization, blinding, intention-to-treat analysis, and outcome measurement to ensure the studies’ internal validity and reliability [27]. For RCT protocols, the SPIRIT checklist was employed to establish standards for transparent and rigorous protocol development [28]. For pilot studies, interventional, and non-randomized experimental studies, the ROBINS-I tool was applied, designed to evaluate the risk of bias in non-randomized studies by addressing variables such as confounding, participant selection, and outcome measurement [29]. Lastly, for qualitative and case studies, the CASP checklist was used, assessing transparency in data collection, the credibility of findings, and researcher reflexivity, which are essential for appropriately interpreting the results in these study types [30,31].

To evaluate the strength of the evidence and the recommendations derived from the results, the Grading of Recommendations Assessment, Development, and Evaluation (GRADE) system was employed. This framework provides a structured approach to rate the quality of evidence, categorizing it as high, moderate, low, or very low. Studies classified as high-quality were those that demonstrated a robust design and execution [32].

To ensure the methodological quality of this systematic review, the guidelines of the PRISMA declaration were strictly followed [33]. 

This systematic review has been registered in the PROSPERO database with the registration number CRD42025630162.

## 3. Results

### 3.1. Search Results

**Figure 2:**
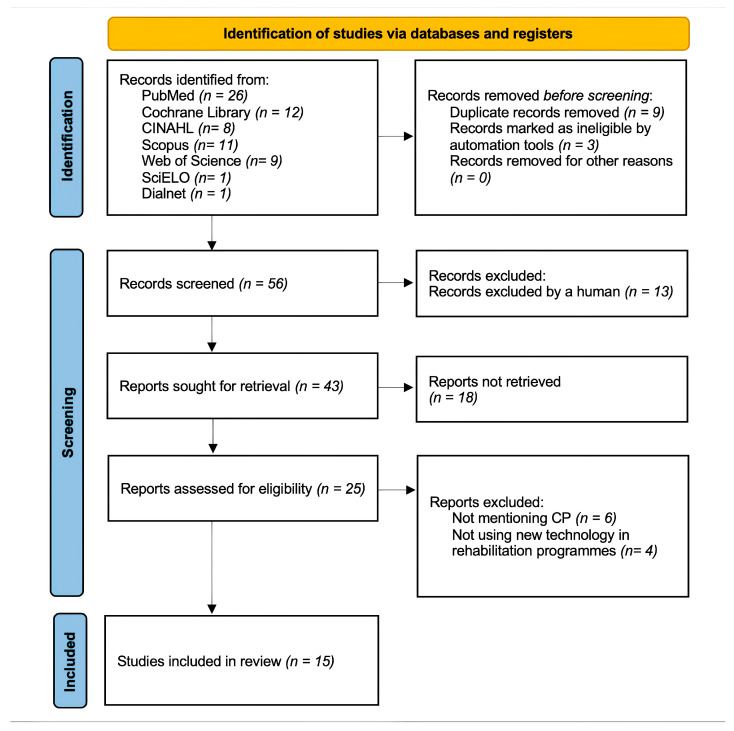
Flow chart.

A total of 68 publications were initially obtained (26 in PubMed, 11 in Scopus, 8 in CINAHL, 12 in Cochrane Library, 9 in Web of Science, 1 in Dialnet, and 1 in SciELO), with 12 being discarded due to duplication, resulting in 56 unique results. A second filter was then applied to exclude reviews, systematic reviews, and meta-analyses, which further reduced the number of publications to 43. Furthermore, 43 articles were screened by the title and/or abstract. A third filter was applied to review the full text completely and to enforce specific selection criteria, and finally, 15 articles were included in this review. These results are depicted in the flow diagram (Figure 2).

### 3.2. Summary of Results

Based on the characteristics of the studies included in this review (Table 1), all participants were children diagnosed with CP. The age range was heterogeneous, including participants aged 4 to 12 years [34,35,36,37,38,39,40,41,42,43,44,45,46,47,48].

The studies applied various technological systems or devices as complementary tools to conventional rehabilitation [34,35,36,37,38,39,40,41,42,43,44,45,46,47,48]. The selected articles demonstrated the use of different devices tailored to CP patients, highlighting the diversity of technological applications in this population [34,35,36,37,38,39,40,41,42,43,44,45,46,47,48].

#### 3.2.1. Lack of Consensus in Protocol Design

There is no consensus among the authors regarding the standardized protocols for implementing technology-based interventions in children with CP to achieve significant results across various domains. Four studies suggest that three sessions per week using different electronic devices can yield positive outcomes [34,35,40,48]. However, other studies report benefits with two sessions [41,42,45] or up to six sessions per week [38]. Similarly, the duration of sessions varies widely, ranging from 40 to 150 min [34,35,38,40,41,42,45,47,48], while protocol durations span from three weeks [34] to 12 weeks [45].

#### 3.2.2. Objectives of Technological Interventions

The included studies utilized technology for various objectives and purposes, as follows:

1. Improving Mobility and Physical Activity
  Five studies focused on the use of assistive technologies and robotic devices to enhance mobility and physical activity in children with CP [34,38,40,45,48]. Interventions included electrical stimulation suits [45], functional electrical stimulation for walking [38], robotic gait training [40], haptic robotic systems with virtual environments [34], and robotic devices for postural support [48]. These interventions aimed to improve motor control and physical activity, enhance independence, and optimize movements to reduce muscle stiffness. For example, Santamaria et al. [48] used a robotic device with rigid postural support to improve body alignment, alleviate muscular tension, and strengthen functionality in children with CP.
2. Enhancing Autonomy in Daily Activities
  Two studies aimed to increase engagement in daily activities and promote autonomy in children with CP. Dalton and Hoyt-Hallett [36] examined how assistive devices, such as wheelchairs, could facilitate participation in environmental activities. Turgeon et al. [46] introduced a mechanical feeding support designed to reduce caregiver dependency and promote self-feeding in children with restricted mobility. These studies highlight the potential of assistive technology to foster autonomy in daily life [36,46]. Additionally, Şahin et al. [41] demonstrated that Virtual Reality (VR) rehabilitation using the Kinect system significantly improved motor skills related to daily tasks, leading to greater autonomy in self-care activities. Chang et al. [42] reported that children receiving VR-based rehabilitation combined with conventional occupational therapy showed enhanced upper limb functionality, which translated into greater independence in ADLs and reduced caregiver assistance. Furthermore, Bono et al. [47] found that the Personalized Upper Limb Intensive Therapy (PULIT) program, integrating goal-directed therapy, bimanual training, and robotic-based exergames, significantly improved dexterity, fine motor skills, and ADL performance, ultimately increasing autonomy and lessening caregiver burden. These studies highlight the potential of assistive technology, VR, and intensive rehabilitation programs to foster independence and functional engagement in children with CP [36,41,42,46,47].
3. Improving Motor Control and Functionality
  Four studies focused on improving motor control in the upper limbs and orofacial musculature using virtual environments and rehabilitation programs [34,39,41,42]. Şahin et al. [41] and Chang et al. [42] demonstrated that VR interventions using the Kinect system and VR-based rehabilitation enhanced upper extremity function in children with unilateral spastic CP and CP, respectively. Martín-Ruiz et al. [39] developed a virtual environment to assess and correct orofacial dysfunctions, while Fluet et al. [34] observed improvements in upper limb motor function, reaching kinematics, and motor control through haptic feedback and virtual environments.
4. Increasing Motivation and Participation in Rehabilitation
  Three studies targeted the improvement of motivation and participation in rehabilitation [35,37,39,41]. Mejía-Rosas et al. [35] evaluated volitional engagement in therapeutic activities using technology-assisted games, aiming to boost motivation. Şahin et al. [41] and Chang et al. [42] reported that VR-based rehabilitation significantly increased patient engagement and participation in therapy. Martín-Ruiz et al. [39] achieved similar goals through virtual environments. Tatla et al. [37] explored the role of technologies like video games and social media in motivating children for upper limb rehabilitation. Despite differences in application, all these interventions shared the goal of enhancing active participation and interest in rehabilitation processes.
5. Impact on Families and Caregivers
  Smethurst et al. [43] emphasized the significance of assistive technology for families and caregivers. Families reported that assistive technologies were essential for enabling children with CP to lead active, meaningful lives and empowering them to control and choose their level of participation in various activities. Similarly, Bono et al. [47] found that intensive upper limb therapy (PULIT) not only improved dexterity and ADL performance but also reduced caregiver burden by enhancing the child’s functional independence.

The studies reviewed demonstrate a broad range of technological applications in CP rehabilitation, each with unique benefits and challenges. While technologies such as robotics, virtual environments, and assistive devices offer promising outcomes in mobility, autonomy, motor control, and motivation, there remains a lack of standardization in intervention protocols. These findings underscore the importance of further research to establish evidence-based guidelines for the effective integration of technology into CP rehabilitation programs.

#### 3.2.3. Classification of Assistive Technologies According to ISO 9999:2022

The new technologies and digital technologies presented in the analyzed studies [34,35,36,37,38,39,40,41,42,43,44,45,46,47,48] have been associated with the ISO 9999:2022 classification to identify the areas where technological advancements have been concentrated.

1. Motor Skills Training (ISO 9999: 04 48 18): Several studies investigated robotic- and VR-based interventions aimed at improving upper limb motor function and gait in children with CP [34,40,41,42,44,47,48]. These technologies align with the motor skills training category by enhancing functional movement and coordination in children with CP.
2. Functional Electrical Stimulation (ISO 9999: 04 36 06): Research on functional electrical stimulation (FES) during daily walking activities in children with spastic CP [38,45] falls within this category. These studies demonstrate a focus on neuromuscular rehabilitation, utilizing FES technology to improve gait and lower limb functionality.
3. Self-Care and Feeding Assistance (ISO 9999: 15 09 03): Assistive devices designed to support self-care and feeding, such as an eating assistance device for individuals with movement disorders [46], align with this category. Additionally, studies by Şahin et al. [41] and Chang et al. [42] showed that VR rehabilitation improved functional independence in self-care activities, such as dressing and eating, reducing caregiver assistance. The PULIT program [47] also emphasized functional self-care improvements, highlighting the role of intensive, goal-directed interventions in enhancing autonomy. Despite these findings, self-care and daily living technologies remain underrepresented in the analyzed literature.
4. Communication and Social Participation Support (ISO 9999: 05 06 12, 22 21 12): Studies exploring technological interventions for social interaction and learning [35,37] correspond to communication aids and training tools for social participation. VR-based interventions indirectly contributed to social participation by enhancing engagement and interaction in rehabilitation settings [44].
5. Postural and Mobility Support (ISO 9999: 12 22, 12 24, 04 48 15): The use of rigid trunk support systems and robotic-assisted gait training [36,40,48] falls into these classifications, emphasizing mobility enhancement. The inclusion of FES [38,45] and robotic exoskeletons further reinforces the focus on postural control and assisted mobility in CP rehabilitation.

The mapping of assistive technologies to ISO 9999:2022 highlights a strong concentration of research in motor skills rehabilitation (ISO 9999: 04 48 18), functional electrical stimulation (ISO 9999: 04 36 06), and mobility support (ISO 9999: 12 22, 12 24, 04 48 15).

VR-based rehabilitation [41,42,44] and intensive motor therapy programs [47] were frequently studied, demonstrating their effectiveness in improving motor function and autonomy.

Conversely, assistive technologies related to self-care (ISO 9999: 09 00) remain underrepresented, indicating a potential research gap in the development of solutions for daily living activities, such as dressing, personal hygiene, and independent feeding.

Additionally, while some studies explored assistive technologies for social participation and communication, these areas received comparatively less attention, suggesting the need for further exploration in CP rehabilitation research.

### 3.3. Level of Evidence and Grade of Recommendation

Different scales were used to assess the risk of bias, methodological quality, level of evidence, and recommendations (Table 2).

For randomized controlled trials [38,40,41,42,44], the PEDro scale was applied. This scale evaluates key aspects such as randomization, blinding, intention-to-treat analysis, and outcome measurement, ensuring the internal validity and reliability of the studies. Based on their PEDro scores, these studies were classified as excellent or good quality with a low risk of bias.

For the randomized controlled trial protocol [48], the SPIRIT checklist was utilized. This tool establishes standards for the development of transparent and rigorous protocols. The study was determined to have a moderate risk of bias, as the protocol addressed many key aspects of the SPIRIT guidelines but showed notable deficiencies in details critical to the completeness and transparency of the study design.

For pilot studies [34,39,45,47] and non-randomized interventional and experimental studies [35], the ROBINS-I tool was employed to evaluate the risk of bias in non-randomized studies, focusing on factors such as confounding variables, participant selection, and outcome measurement. All these studies exhibited a moderate risk of bias due to insufficient information regarding participant selection criteria, handling of missing data, and potential deviations from the intervention protocol.

For qualitative studies [37,43,46] and case reports [36], the CASPe checklist was used. This scale assesses transparency in data collection, the credibility of findings, and researcher reflexivity, which are essential for accurately interpreting results in these study types. The studies by Smethurst et al. [43], Tatla et al. [37], and Turgeon et al. [46] demonstrated high methodological quality, whereas the study by Dalton and Hoyt-Hallett [36] showed moderate methodological quality with some limitations.

After evaluating the methodological quality of the studies, the level of evidence and strength of recommendations based on the results were assessed using the Grading of Recommendations Assessment, Development, and Evaluation (GRADE) framework. This system provides a structured approach to categorizing evidence quality as high, moderate, low, or very low [24]. Studies classified as high quality were those that were well designed and executed. The results of these assessments are presented in Table 2.

## 4. Discussion

Emerging technologies have demonstrated significant potential as effective tools for physical training, fostering both motor and cognitive improvements across various populations [49]. This systematic review aimed to analyze the available data on the application of innovative technologies in occupational therapy for the treatment of pediatric CP. It sought to understand how these technologies impact patients and determine their contribution to improving autonomy.

The review employed a rigorous methodology to analyze the scientific evidence available for CP rehabilitation programs, including assessments of evidence levels and strength of recommendations. The selected studies encompassed a variety of technological applications and protocols, evaluating their impact on a range of health-related outcomes. This comprehensive approach provides valuable insights into the potential of emerging technologies to enhance rehabilitation outcomes in pediatric CP.

This systematic review highlights the potential of emerging technologies and assistive devices in the rehabilitation of children with CP, demonstrating significant progress in addressing motor deficits [34,38,39,40,41,42,44,45,48], enhancing functional autonomy, and promoting participation in daily activities [35,36,37,38,41,42,46]. However, the studies also reveal limitations that must be addressed to optimize their clinical implementation and identify areas requiring further exploration.

One of the key benefits observed in the analyzed studies is the effectiveness of robotic and VR technologies in motor rehabilitation for both upper and lower extremities, as well as their ability to enhance mobility and motor function in patients with CP. This positive impact is evidenced by interventions such as the electrical stimulation suit described by Flodström et al. [45] and robotic-assisted gait training for improving locomotion, as studied by Ammann-Reiffer et al. [40], both of which demonstrated efficacy in improving gait and motor function [40,45]. Furthermore, Pool et al. [38] highlighted the utility of FES in optimizing motor performance during daily activities, such as walking. Similarly, Fluet et al. [34] demonstrated how integrating haptic robotic systems with complex virtual environments can improve upper limb motor functions in children with CP. The effectiveness of VR-based rehabilitation has been further supported by studies such as Şahin et al. [41], Chang et al. [42], and Choi et al. [44], which demonstrated significant improvements in upper limb motor functions, functional independence, and engagement in rehabilitation. Şahin et al. [41] found that VR rehabilitation using the Kinect system significantly improved gross and fine motor skills, translating into better ADL performance. Similarly, Chang et al. [42] showed that children receiving VR therapy combined with conventional OT experienced greater upper limb functionality, leading to improved autonomy. Choi et al. [44] expanded on these findings, demonstrating that VR interventions enhance neuroplasticity and promote motor learning in children with brain injuries, reinforcing the benefits of immersive rehabilitation environments.

These technological approaches not only provide a controlled and safe environment for repetitive practice but also foster motivation and participant engagement. The findings underscore the value of these technologies as tools for enhancing motor capacities and improving the quality of life for patients with CP. These results align with research in other areas, such as Parkinson’s disease [50], multiple sclerosis [51], and cardiac rehabilitation [16], where the use of technology in motor rehabilitation—particularly for gait training—has been shown to be highly effective and widely adopted.

Another significant finding is the positive impact of these technologies on ADLs and the quality of life of patients. Turgeon et al. [46] explored assistive devices for feeding in children with CP, while Ryan et al. [52] analyzed the benefits of adaptive seating to enhance functionality in this population. Both studies highlighted how occupational therapy interventions can improve autonomy and participation in essential tasks such as feeding and mobility. Similarly, Şahin et al. [41], Chang et al. [42], and Bono et al. [47] provided strong evidence that technology-based rehabilitation improves functional independence in daily activities. Şahin et al. [41] demonstrated that VR interventions enhance engagement in ADLs, while Chang et al. [42] showed that children undergoing VR therapy exhibited reduced reliance on caregivers. Bono et al. [47] further emphasized the role of intensive upper limb therapy in improving dexterity and self-care skills, leading to greater autonomy and caregiver relief. Notably, Turgeon et al. [46] emphasized that technological interventions not only address physical limitations but also promote independence and social integration in children with CP. Komariah et al. [53] further noted the positive impact of VR on ADLs in children with CP. VR facilitates the recreation of functional and practical scenarios within controlled environments, enabling the development of motor and functional skills necessary for performing everyday activities. This capability is indispensable in rehabilitation processes, as improvements in ADLs directly contribute to the autonomy and quality of life of children, making VR an essential component of effective occupational therapy programs. However, as Smethurst et al. [43] highlighted, it is crucial to involve parents in the decision-making process and tailor interventions to the individual needs of each child. This underscores the importance of designing family-centered rehabilitation programs that are adapted to the specific characteristics of each patient, ensuring that therapeutic goals align with their unique circumstances.

Emerging technologies, such as software applications, video games, and VR, present an exciting opportunity to expand intervention strategies for CP. These tools are becoming increasingly prominent in healthcare due to their ability to provide a more functional and dynamic approach to rehabilitation. Martín-Ruiz et al. [39] highlighted the potential of virtual environments to create innovative possibilities for functional recovery. Similarly, Tatla et al. [37] emphasized that interactive technologies and video games offer an engaging approach that could enhance participation in rehabilitation programs for children with CP. This level of interaction is crucial for fostering commitment and adherence to therapy. Despite these promising advancements, further research is needed to explore how these tools can be effectively integrated with conventional therapies and to evaluate their long-term clinical efficacy. Moreover, these technologies enable more precise and personalized assessments, such as the early identification of oral or motor dysfunctions, which could further enhance the effectiveness of interventions [37,39].

Several studies highlight that low-cost gaming consoles are among the most commonly used technologies in rehabilitation programs due to their accessibility and ease of implementation [16,49,51,54]. According to Tatla et al. [37], while these tools are viewed positively by therapists for upper limb rehabilitation, their effective integration into therapeutic programs requires careful design to avoid inefficacy. This finding underscores the need for protocols that balance technological innovation with practical and clinical application. However, low-cost consoles are neither the only technological tools available nor necessarily the most effective in all contexts. The reviewed literature does not identify a clear consensus on which type of technology yields the best results [34,35,36,37,38,39,40,41,42,43,44,45,46,47,48]. Regarding specific assistive devices, benefits have been reported in various functional areas. For instance, Pool et al. [38] found that daily functional electrical stimulation significantly improved satisfaction and performance in walking activities for children with unilateral spastic CP. Similarly, innovative technologies like the Mollii^®^ suit, evaluated by Flodström et al. [45], demonstrated improvements in body function and social participation, although its clinical application remains in preliminary stages. These interventions highlight the importance of tailoring technologies to the specific needs of each patient to maximize their effectiveness. This raises the necessity of carefully evaluating the accessibility and feasibility of technologies before their widespread integration into clinical treatments. These findings emphasize the importance of continued research to identify which technologies are most appropriate based on therapeutic objectives and the individual conditions of patients. This lack of consensus may be attributed to the heterogeneity in study designs, limited descriptions of the characteristics of children with CP, and the varying needs of patients depending on their specific conditions. Furthermore, from an economic perspective, although emerging technologies offer promising benefits, their high cost poses a significant challenge for both families and healthcare systems [43]. While the analyzed studies provide valuable insights into the effects of new technologies on CP patients, they also reveal several limitations that should be considered when interpreting the results [34,35,36,37,38,39,40,41,42,43,44,45,46,47,48].

An important methodological concern is the lack of blinding among therapists in some studies [34,35,36,39,45,46]. This could have influenced how interventions were delivered or outcomes assessed, introducing bias that compromises the internal validity of the research. For instance, the therapist’s perception of the intervention’s effectiveness may affect both the administration of treatment and the interpretation of results. Such bias must be carefully considered to enhance the reliability and credibility of the findings [32].

Another significant limitation is the management of confounding factors and missing data. Not all studies specify how variables such as the severity of CP or the presence of comorbidities are controlled, both of which can substantially influence the final outcomes. Furthermore, the inclusion and exclusion criteria are not always clearly defined, increasing the risk of selection bias and raising concerns about the representativeness of the samples. Inaccurate reporting of follow-up losses also undermines the validity of the results, introducing uncertainty regarding the consistency of the analyzed data [32]. In conclusion, emerging technologies and assistive devices present significant opportunities to enhance rehabilitation outcomes in children with CP. However, further research is essential, involving larger sample sizes and controlled study designs, with a focus on evaluating their long-term efficacy and economic accessibility. Moreover, a patient- and family-centered approach is required to ensure that these tools are tailored to meet the specific needs of each individual. Striking a balance between technological innovation and practical application will be critical to maximizing the benefits of these interventions in pediatric CP rehabilitation.

This systematic review has several limitations that should be taken into account when interpreting the results. First, access to information on this topic is limited due to a scarcity of publications, emphasizing the need for more research employing rigorous and high-quality methodologies. Furthermore, the review was restricted to articles published in Spanish and English and those available as open access, which may have excluded relevant studies published in other languages or formats with restricted access. Lastly, many of the included studies were pilot studies or had small sample sizes, limiting the generalizability of their findings and reducing statistical power. To advance this field, it is essential to conduct studies with larger sample sizes and controlled designs that allow for the evaluation of the long-term efficacy of these technologies and their seamless integration into existing treatments. Moreover, it is crucial to enhance current technologies by optimizing their design and functionality to maximize their benefits and ensure their effective implementation in clinical practice. These limitations highlight the need for continued research to strengthen the evidence on the impact of emerging technologies on patients with CP and to address the methodological and practical challenges that persist in this area.

## 5. Conclusions

This systematic review highlights the diverse applications of emerging technologies in occupational therapy for children with CP, including assistive robotics, virtual environments, and functional electrical stimulation. These technologies show potential benefits in mobility, motor control, autonomy, and motivation. These improvements contribute to enhancing quality of life. However, a lack of standardized intervention protocols and variability in methodological quality limit the generalizability of findings. In conclusion, the analysis of the reviewed studies suggests that emerging technologies have significant potential to enhance the rehabilitation of children with CP, improving motor abilities, fostering social interaction, and facilitating engagement in ADLs.

While motor skill training and functional electrical stimulation are well represented, self-care and social participation technologies remain underexplored, indicating a research gap. Despite positive outcomes, further high-quality randomized controlled trials and standardized protocols are needed to strengthen evidence and optimize clinical integration. Future research should focus on expanding technological applications to enhance autonomy and quality of life in children with CP.

However, it is essential to further investigate their effectiveness through methodologically robust studies with larger sample sizes to refine their application and ensure their adaptability across diverse populations.

## Figures and Tables

**Table 1:** Characteristics of the articles included.

Author	Year	Type of Study	Age (Years)	Patients	Aim	Type of Technology	Intervention Groups	Protocol	Results
Fluet et al. [34]	2010	Pilot study	9	9	To investigate how training with the New Jersey Institute of Technology Robot-Assisted Virtual Rehabilitation (NJIT-RAVR) system can induce changes in upper limb function in children with hemiplegia caused by CP.	The New Jersey Institute of Technology Robot Assisted Virtual Rehabilitation (NJIT-RAVR) system.	- Study group one (NJIT-RAVR sessions) - Study group two (NJIT-RAVR sessions) - Study group three (NJIT-RAVR plus CIMT and intensive bimanual therapeutic interventions).	3 sessions per week and each lasting 60 min, for 3 weeks, for a total of 9 sessions in three groups.	The results indicate significant increases in active supination achieved during training, which translated into improvements in active range of motion during post-testing. Participants demonstrated notable enhancements in active range of motion measurements, upper limb motor function, reaching kinematics, and motor control.
Mejía-Rosas et al. [35]	2010	Observational prospective, opened, case–control study with related samples	10	12	To evaluate the level of volition during gameplay using a switch adapted to a computer mouse in children with moderate spastic CP.	A modified environment utilizing assistive technology through an adapted mouse and various activities was implemented.	- A single group received intervention using assistive technology.	3 sessions per week and each lasting 40 min, for 4 weeks, for a total of 10 sessions in three groups.	The results indicate that children developed increased interest and willingness to participate in gameplay, enhancing their social integration. The findings demonstrate that modifying the environment in which children with moderate spastic CP interact significantly improves their volition to engage in play activities, thereby facilitating a direct connection between the child and the computer.
Dalton and Hoyt-Hallett [36]	2013	Case reports	12	2	To explore, through case reports, the occupational deprivation experienced by two preadolescent girls with CP and the role of assistive technology in facilitating occupational engagement and addressing occupational injustices.	VMax^®^ communication device with Picture WordPower^®^ software.	- Case study of two unique cases using the VMax^®^ communication device with Picture WordPower^®^ software.	Two years.	Findings suggest that the use of assistive technology can significantly reduce occupational deprivation and social exclusion in children with physical disabilities. Participation enabled through technology expanded the children’s social networks and facilitated the establishment of meaningful relationships. Additionally, access to appropriate assistive technology transformed the relationship between the user and her mother, fostering improved communication and interaction.
Tatla et al. [37]	2015	Qualitative research	25–65	10 (4 occupational therapists and 6 physiotherapists)	The aim of our study was to examine therapists’ perspectives on the use of gaming and social media technologies by young people and adults with hemiplegia in their daily lives and rehabilitation practices, as well as to identify the obstacles to incorporating these technologies into rehabilitation.	Social media and video gaming for therapy.	- 2 focus groups with a semi-structured focus group guide.	Each focus group of between 4 and 6 participants lasted 90 min.	Preliminary research on commercial gaming indicates potential benefits; however, larger and more robust randomized clinical trials are necessary to fully establish the clinical efficacy of these gaming interventions. This study highlights the nuanced challenges involved in integrating new technologies into clinical practice. Therapists recognized the potential for these tools to enhance social interactions.
Pool et al. [38]	2015	Randomized clinical trial	10.8 ± 3.3	32	To evaluate whether daily functional electrical stimulation is effective in improving the perception of children with unilateral spastic CP regarding individually identified mobility challenges.	The Walk Aide^®^ device.	- FES treatment group (The Walk Aide^®^ device). - Control group (usual orthotic protocol).	6 sessions per week and each lasting 240 min, for 8 weeks, for a total of 48 sessions in two groups.	Daily application of Functional Electrical Stimulation through the Walk Aide^®^ device during routine walking effectively addresses self-identified priorities by improving both the performance and satisfaction with functional skills following treatment.
Martín-Ruiz et al. [39]	2016	Pilot study of assistive technology development	4–10	17	The primary objective of this validation test was to evaluate how children with PCP responded to the SONRIE games and to compare their outcomes with those obtained during the verification phase.	SONRIE System (uses Microsoft’s Kinect sensor connected to a PC).	- Experimental group (children with PCP using the SONRIE system). - Control group (healthy children using the SONRIE system).	2 sessions. They established a maximum time limit of 15 s and required each exercise to be performed three times, during which the child was expected to execute the movement.	SONRIE, a proposed technological system, has the potential to enhance facial muscle function, contributing to an improved quality of life for children and their families. It utilizes interactive games as therapeutic tools, enabling personalized therapies and mitigating the effects of CP in children. The validation of SONRIE demonstrates its effectiveness in improving the detection and intervention of orofacial muscle function, supporting its use in rehabilitation practices.
Ammann-Reiffer et al. [40]	2017	Randomized clinical trial	Not reported	Not reported	To examine the impact of robotic-assisted gait training on improving functional gait parameters in children with CP.	Robot-assisted gait training with the Lokomat	- T/C group (RAGT plus usual care) - C/T/C group (usual care plus RAGT plus usual care)	T/C group: 5 weeks of RAGT (3 sessions per week and each lasting 45 min) plus 5 weeks of usual care (1–2 sessions per week), for a total of 11 weeks. C/T/C group: 5 weeks of usual care plus 5 weeks of RAGT plus 5 weeks of usual care, for a total of 16 weeks.	While robotic-assisted gait training has become an established treatment option for addressing gait disorders, evidence supporting its efficacy remains inconclusive. This study aims to provide critical insights into its effects under clinical ambulatory conditions.
Şahin et al. [41]	2020	Single-blind randomized controlled trial	10.28 ± 3.43	60	To investigate the effects of VR using Kinect on gross and fine motor functions, as well as independence in ADLs in children with unilateral spastic CP.	VR-based intervention using the Kinect system.	- VR intervention group (VR with video games through the Kinect system). - Traditional occupational therapy group (motor skills and daily activities, such as dressing, feeding, playing table and ball games, writing, painting, drawing, and doing puzzles).	2 sessions per week and each lasting 45 min, for 8 weeks, for a total of 16 sessions.	Both groups showed significant improvements in overall motor functions and independence in ADLs after the 8-week intervention. However, the VR group showed significantly greater improvements in gross and fine motor functions as well as daily activities compared to the TOT group. This study suggests that VR-based intervention with Kinect is effective in improving motor functions and independence in ADLs in children with unilateral spastic CP.
Chang et al. [42]	2020	Retrospective study	4–6	17	To evaluate the effects of VR-based rehabilitation combined with conventional occupational therapy on upper limb function and caregiver assistance in children with CP.	RAPAEL Smart Kids device and video games.	- Smart Glove group (VR rehabilitation combined with conventional OT). - Conventional occupational therapy group (conventional OT focused on upper limb function).	2 sessions per week and each lasting 30 min, for 8 weeks, for a total of 16 sessions.	This study suggests that VR-based rehabilitation combined with conventional OT can improve upper limb function and reduce caregiver burden in children with CP. These enhancements in upper extremity function, combined with repeated practice of ADLs, have led to a significant reduction in caregiver burden.
Smethurst et al. [43]	2021	Qualitative research	Not reported	8	This research aimed to examine the experiences of families with a child with PCP after 12 months of receiving support through the National Disability Insurance Scheme (assistive technology), compare their perspectives to previous funding systems, and provide recommendations for the future development of the National Disability Insurance Scheme.	Assistive technology.	- 8 Semi-structured interviews.	Semi-structured interviews ranged from 60 to 90 min.	Participants reported significant delays in accessing assistive technology, which had a profound impact on their daily lives. Their interactions with the National Disability Insurance Scheme (NDIS) were described as frustrating due to administrative challenges, communication issues, and a lack of personalized approaches. Nevertheless, all participants recognized assistive technology as essential for enabling their children to lead active and meaningful lives, as well as empowering them to control and select their level of participation in various activities.
Choi et al. [44]	2021	Randomized controlled trial	3–16	80	This study aimed to assess the effectiveness of a VR-based rehabilitation system in enhancing upper limb function in children with brain injury, compare its outcomes to conventional occupational therapy, and provide insights for the future integration of VR technology in pediatric rehabilitation programs.	VR with wearable inertial sensors.	- VR group (30 min of VR-based therapy + 30 min of conventional OT). - Control group (60 min of conventional OT).	5 sessions per week and each lasting 60 min, for 4 weeks, for a total of 20 sessions.	Both groups showed significant improvements compared to baseline. The VR group demonstrated greater improvements in upper limb dexterity, ADLs, and forearm supination. Children with greater motor impairment benefited more from VR therapy. The study concluded that VR-based rehabilitation is an effective, engaging, and intensive motor training tool for children with brain injury (including CP).
Flodström et al. [45]	2022	Pilot study	7.7	6	The study assessed the potential effects of Mollii^®^ on body function, activity, and participation in self-selected activities.	Device electro-suit Mollii^®^.	- Experimental group (children with PCP using Mollii^®^.	2 sessions per week and each lasting 60 min, for 12 weeks, for a total of 24 sessions.	All participants demonstrated improvements in their total scores on the Canadian Occupational Performance Measure. Additionally, pain levels decreased among children who reported pain at baseline. The Mollii^®^ electro-suit showed a positive impact on both performance and comfort. However, further studies with larger sample sizes and longer follow-up periods are necessary to confirm these findings and evaluate long-term effects.
Turgeon et al. [46]	2022	Qualitative research	>6	11	To develop and present a prototype assistive device designed to support individuals with movement disorders, such as CP, in achieving greater independence during feeding. Additionally, a preliminary evaluation was conducted to assess the device’s performance and guide the development of future iterations.	Device to assist with eating, aimed at stabilizing the movement of people who have movement disorders.	- A questionnaire was designed to evaluate user satisfaction with the technological device, assess user needs, and determine the relevance of the new device in providing assistive support for feeding. - Focus group with occupational therapists.	Not reported.	This fully mechanical device stabilizes utensils while compensating for the user’s movements. It was observed to improve overall posture by helping maintain the optimal alignment of the arm and body to reach the spoon. In the short term, efforts will focus on refining the prototype using data collected during the study. In the future, a new version of the device is planned, incorporating smart electronic dampers to enhance its functionality.
Bono et al. [47]	2022	Retrospective analysis	7.8 ± 2.1	23	To determine the effectiveness of the PULIT program in helping children with hemiparesis achieve individual goals and transfer motor skills to ADLs.	PULIT program plus robotics-based exergames (YouGrabber System, Armeo Spring, Diego, and Myro).	- Experimental group (a structured approach combining modified Constraint-Induced Movement Therapy (mCIMT), Bimanual Intensive Therapy (BIT), and exergame-based robotics).	4 sessions per week and each lasting 150 min, for 2 weeks, for a total of 8 sessions.	The PULIT program effectively enhances dexterity in children with CP in both the affected and dominant upper limb, improves performance in ADLs, and facilitates the achievement of individual goals. This article presents an OT program that integrates technology-based and rehabilitation interventions, enabling children with upper limb hemiparesis to improve relevant daily tasks within just 8 days.
Santamaria et al. [48]	2023	Study protocol for a randomized controlled trial.	Not reported.	82	To compare the efficacy of the TruST robotic system, which provides dynamic postural support, with a static trunk support system.	Robotic Trunk-Support-Trainer (TruST)	- Experimental group (Trunk-Support-Trainer intervention). - Control group (Static Trunk-Support intervention).	3 sessions per week and each lasting 120 min, for 4 weeks, for a total of 12 sessions.	The use of the TruST system is expected to improve posture and reaching capabilities, with its force field technology anticipated to further enhance these benefits. While participants may experience physical fatigue, no significant safety concerns are anticipated.

**Table 2:** Risk of bias, methodological quality, level of evidence, and recommendation.

	PEDro	SPIRIT	ROBINS-I	CASPe	GRADE
Fluet et al. [34]	-	-	Moderate risk of bias	-	Low (++)
Mejía-Rosas et al. [35]	-	-	Moderate risk of bias	-	Low (++)
Dalton and Hoyt-Hallett [36]	-	-	-	Moderate quality	Low (++)
Tatla et al. [37]	-	-	-	High quality	Low (++)
Pool et al. [38]	Low risk of bias. Excellent quality. 10/10	-	-	-	High (++++)
Martín-Ruiz et al. [39]	-	-	Moderate risk of bias	-	Low (++)
Ammann-Reiffer et al. [40]	Low risk of bias. Excellent quality. 9/10	-	-	-	High (++++)
Şahin et al. [41]	Low risk of bias. Good quality. 6/10	-	-	-	High (++++)
Chang et al. [42]	Low risk of bias. Good quality. 6/10				High (++++)
Smethurst et al. [43]	-	-	-	High quality	Low (++)
Choi et al. [44]	Low risk of bias. Good quality. 6/10	-	-	-	High (++++)
Flodström et al. [45]	-	-	Moderate risk of bias	-	Low (++)
Turgeon et al. [46]	-	-	-	High quality	Low (++)
Bono et al. [47]	-	-	Moderate risk of bias	-	Moderate (+++)
Santamaria et al. [48]	-	Full compliance	-	-	High (++++)
